# Comparative analysis of the fecal bacterial community of five harbor seals (*Phoca vitulina*)

**DOI:** 10.1002/mbo3.369

**Published:** 2016-05-14

**Authors:** Daniela Numberger, Daniel P. R. Herlemann, Klaus Jürgens, Guido Dehnhardt, Heide Schulz‐Vogt

**Affiliations:** ^1^ Leibniz Institute for Baltic Sea ResearchSeestrasse 15RostockD‐18119Germany; ^2^ University of RostockAlbert‐Einstein‐Strasse 3RostockD‐18059Germany; ^3^ Marine Science CenterAm Yachthafen 3aRostockD‐18119Germany; ^4^ Present address: Leibniz Institute for Zoo and Wildlife ResearchAlfred‐Kowalke‐Strasse 17D‐10315Berlin

**Keywords:** Gut bacteria, microbial ecology, microbiome.

## Abstract

The gut microbiota has many beneficial effects on host metabolism and health, and its composition is determined by numerous factors. It is also assumed that there was a co‐evolution of mammals and the bacteria inhabiting their gut. Current knowledge of the mammalian gut microbiota mainly derives from studies on humans and terrestrial animals, whereas those on marine mammals are sparse. However, they could provide additional information on influencing factors, such as the role of diet and co‐evolution with the host. In this study, we investigated and compared the bacterial diversity in the feces of five male harbor seals (*Phoca vitulina*). Because this small population included two half‐brother pairs, each sharing a common father, it allowed an evaluation of the impact of host relatedness or genetic similarity on the gut microbial community. Fresh feces obtained from the seals by an enema were analyzed by fluorescence in situ hybridization and amplicon sequencing of 16S rRNA genes. The results showed that the bacterial communities in the seals' feces mainly consisted of the phyla *Firmicutes* (19–43%), *Bacteroidetes* (22–36%), *Fusobacteria* (18–32%), and *Proteobacteria* (5–17%) . Twenty‐one bacterial members present in the fecal samples of the five seals contributed an average relative abundance of 93.7 + 8.7% of the total fecal microbial community. Contrary to all expectations based on previous studies a comparison of the fecal community between individual seals showed a higher similarity between unrelated than related individuals.

## Introduction

The gut bacterial community of mammals plays an important role in host health by providing, for example, an additional source of energy via the fermentation of otherwise indigestible carbohydrates (e.g., Bergman et al. 1965; Parker 1976; Turnbaugh et al. 2006) and a supply of vitamins (e.g., Ramotar et al. 1984; Gill et al. 2006), and by contributing to the development of the host immune system (e.g., Cebra 1999; Mazmanian et al. 2005; Ivanov et al. 2008). The gut microbiota also constitutes a natural barrier to colonization by pathogenic microbes, a phenomenon referred to as “colonization resistance” (Van der Waay et al. 1971; Van den Bogaard et al. 1986; Gorbach et al. 1988; He et al. 2010). In return, the mammalian host supplies the bacterial community with nutrients and a stable environment (Leser and Molbak 2009). This symbiotic relationship suggests the co‐evolution of mammals and bacteria (Ley et al. 2008a,b). In addition to the fitness advantage conferred on the host by these beneficial microorganisms, their host–host transmission might be facilitated by both parental care and social behavior (e.g., Troyer 1984; Nalepa et al. 2001; Ley et al. 2005; Lombardo 2008).

Recent studies on the gut microbiota of humans and mammals have identified many host factors that influence bacterial community composition. They include the composition and type of diet (e.g., Castillo et al. 2007; Ley et al. 2008a; De Filippo et al. 2010), genotype (e.g., Zoetendal et al. 2001; Benson et al. 2010; Kovaks et al. 2011), gut morphology and physiology (e.g., Ley et al. 2008a; Nelson et al. 2013), social interactions (e.g., Thompson et al. 2008; Bailey et al. 2011; Nelson et al. 2013a), health and weight (e.g., Zhang et al. 2008; Turnbaugh et al. 2009; Claesson et al. 2012), and antibiotic exposure (e.g., Ambrose et al. 1985; Dethlefsen et al. 2008; Dethlefsen and Relman 2011). Animals studies have suggested that the gut microbiota is more similar in conspecifics than in hosts of different species and that host phylogeny is reflected in the composition of the gut microbiota (e.g., Ley et al. 2008a,b; Yildirim et al. 2010). However, this knowledge has been obtained primarily from investigations of humans or terrestrial animals, whereas less is known about the gut microbiota of marine mammals and the factors that influence it. Pinnipeds (walruses, fur seals, sea lions, and true seals), which differ from other marine mammals in their amphibious way of life, are mainly piscivorous; thus, their diet is high in proteins and polyunsaturated fatty acids (Hume et al. 2004), which is likely to be reflected in a gut microbiota whose composition differs from that of other (terrestrial) carnivorous mammals (e.g., Ley et al. 2008a,b; Nelson et al. 2013a).

In their study of nine wild hooded seals (*Cystophora cristata*), one wild harbor seal (*Phoca vitulina*), and one wild gray seal (*Halichoerus grypus*), Glad et al. (2010) used 16S rRNA gene clone libraries and cultivation to evaluate the bacterial diversity and ampicillin and tetracycline resistances of isolates from the colon contents of these animals. Nelson et al. (2013a) studied Antarctic populations of wild southern elephant seals (*Mirounga leonina*) and leopard seals (*Hydrurga lep tonyx*) as well as two captive leopard seals. They suggested that diet, gut length and physiology, social interactions, captivity, sex, age, and species determine the bacterial composition of the host gut microbiota. The authors also concluded that in these animals, bacterial core members are transferred vertically from mothers to pups and may be conserved in the host phylogeny. Smith et al. (2013) studied 21 Australian fur seals (*Arctocepahlus pusillus doriferus*) and found age‐related differences in the composition of their gut microbiota. In the study of Lavery et al. (2012), the microbial metagenome data of feces from one Australian sea lion (*Neophoca cinerea*) were linked to high nutrient transport and cycling potential dominated by the core metabolic functions of carbohydrate utilization, protein metabolism, and DNA metabolism.

The aim of our study was to add further data on the gut microbiota of pinnipeds. Specifically, we first determined the bacterial diversity in the feces of five male harbor seals (*Phoca vitulina*) living a semi‐natural lifestyle within a fenced‐in area of the Baltic Sea. This small population includes two half‐brother pairs, each sharing a common father. Due to the tameness of the animals, it was possible to obtain fresh feces from living healthy pinnipeds, via an enema administered to the animals. We then examined their relatedness or genotype as influencing factor. The absence of a common mother was an advantage, since the first bacterial colonization of the mammalian gut occurs during passage of the neonate through the birth canal, which might obscure the effect of relatedness (e.g., Bettelheim et al. 1974; Long and Swenson 1977; Mändar and Mikelsaar 1996; Ley et al. 2005).

## Material and methods

### Subjects and collection of samples

Fecal samples were taken once from five of eight male harbor seals from the Marine Science Center in Rostock, Germany, where they live in a fenced‐in area of the Baltic Sea. Thus, the seals have access to free‐living fish and crustacean species, but they are also fed daily with sprats and herring. Two seals have not been sampled due to illness and antibiotic treatment and one was not familiar with procedure of an enema. Fecal material was obtained by an enema, administered to the animals during a medical examination out of the water, as previously described (Staniland and Taylor 2003). Briefly, a 1.5‐L enema bag (B. Braun Melsungen AG, Germany) was filled with approximately 1 L of sterile‐filtered prewarmed water and connected to a rectal tube (Ø 6.7 mm, Ratiomed, Germany). The water was introduced into the animal′s colon via the anus. Fecal material was expelled naturally by the animal within 10 min and collected immediately into 50‐mL polyethylene tubes. The samples were placed on ice during their transport to the laboratory, where they were frozen at −80°C until use (Mueller et al. 2006).

### Fluorescence in situ hybridization (FISH)

Cy3‐labeled 16S rRNA oligonucleotide probes (Table 1) were selected from the probeBase website (http://www.microbial-ecology.net/probebase/default.asp *,* Loy et al. 2007) after referring to the literature (e.g., Smith et al. 2013). The probes were checked for their specificity using Probe Match, via the RDP website (http://rdp.cme.msu.edu/index.jsp), and commercially synthesized by Biomers, Germany. Fecal samples were prepared and fixed as described previously (Franks et al. 1998; Mueller et al. 2006). Briefly, fresh fecal material (0.6–1.2 g) was diluted 10‐fold with 1 × phosphate‐buffered saline. After the addition of 5–10 glass beads (diameter 3 mm) to the sample, it was vortexed until the fecal material had decomposed. Centrifugation of the sample at 300*g* for 5 min to remove debris was followed by the transfer of 1 mL of the supernatant into a new polyethylene tube and an overnight fixation at 4°C in 3 mL of 4% paraformaldehyde (v/v). The sample was then mixed thoroughly, divided into four 1‐mL aliquots, and stored at −80°C.

**Table 1:** Cy3‐labeled 16S rRNA oligonucleotide probes used for fluorescence in situ hybridization (FISH). The probes were selected from the probeBase website (http://www.microbial-ecology.net/probebase/default.asp, (Loy et al. 2007)) and synthesized by Biomers, Germany

Name	(Sequence 5′→3′)^a^	Target group^b^	Reference
ATO291	GGT CGG TCT CTC AAC CC	*Atopobium* cluster	Harmsen et al. 2000;
BAC303	CCA ATG TGG GGG ACC TT	*Bacteroidaceae/Prevotellaceae*	Manz et al. 1996;
Erec482	GCT TCT TAG TCA RGT ACC G	*Clostridium* cluster XIVa+b	Franks et al. 1998;
Ent	CCC CCW CTT TGG TCT TGC	*Enterobacteriaceae*	Kempf et al. 2000;
FUS664	CTT GTA GTT CCG CYT ACC TC	*Fusobacterium*	Thurnheer et al. 2004

a Sequence in IUPAC code: R = G/A, Y = T/C, M = A/C, K = G/T, S = G/C, W = A/T, H = A/C/T, B = G/T/C, V = G/C/A, D = G/A/T, N = G/A/T/C.

b Probe specificity was determined by checking the probe sequences against database sequences using Probe Match, via the RDP website (http://rdp.cme.msu.edu/index.jsp), and the following search options: 0 mismatches; type and non‐type strains; source: uncultured and isolates; size: <1200 nucleotides and >1200 nucleotides; good quality.

The thawed fecal samples were filtered on 47‐mm polycarbonate membrane filters (0.2–*μ*m pore size, Whatman). The filters were then cut into sections, so that there was one for each oligonucleotide probe. Hybridization of the bacteria with the membrane filters was performed as described previously, with slight modifications (Glöckner et al. 1996). The filter section for hybridization with the ATO291 probe was first incubated with lysozyme (10 mg mL^−1^) and then with achromopeptidase (60 U mL^−1^), each for 10 min at room temperature (Sekar et al. 2003). A quantity of 2 mL of hybridization buffer [formamide concentration depending on the probe, 0.9 mol L^−1^ NaCl, 20 mmol L^−1^ Tris‐HCl (pH 7.5), 0.01% w/v sodium dodecyl sulfate (SDS), distilled water] was prepared. For each filter section, 19 *μ*L of hybridization buffer was mixed with 1 *μ*L of Cy3‐labeled 16S rRNA oligonucleotide probe (final concentration 2.28 pmol *μ*L^−1^) and then dripped onto Parafilm‐covered glass slides. The filter sections were placed cell‐side down on the hybridization mix, after which the slide was placed horizontally in a 50‐mL polyethylene tube containing a piece of blotting paper soaked with the remaining hybridization buffer. Hybridization with the probes ATO291 (Harmsen et al. 2000) and Erec482 (Franks et al. 1998) was carried out at 50°C for 16 h. For probes BAC303 (Manz et al. 1996), Ent (Kempf et al. 2000), and FUS664 (Thurnheer et al. 2004), the incubation conditions were 46°C for 1.5 h (BAC303, Ent) or 3 h (FUS664). The hybridized filters were then washed for 30 min in preheated washing buffer [NaCl concentration depending on the formamide concentration in the hybridization buffer, 20 mmol L^−1^ Tris‐HCl (pH 8.0), 5 mmol L^−1^ EDTA (pH 8.0), 0.01% SDS, distilled water], at 48°C (BAC303, Ent, FUS664) or 50°C (ATO291, Erec482), then for 1 min in distilled water and 1 min in 96% ethanol. The air‐dried filter sections were mounted in a 1:5 (v/v) Vecta Shield‐Citifluor mix containing 1 *μ*g DAPI mL^−1^ for counterstaining. The slides with the mounted filters were stored in the dark at 4°C.

Hybridized and 4',6‐diamidino‐2‐phenylindole (DAPI)‐stained cells were counted directly using an epifluorescence microscope (Axioskop2 MOT Plus; Zeiss, Germany) and the 02 filter set (488002‐9901‐000) for DAPI or the 15 filter set (488015‐0000‐000) for the Cy3‐labeled probes (Zeiss, Germany). The cells in at least ten optical fields per filter fragment and probe were counted.

### DNA extraction and PCR

Total genomic DNA was extracted from fecal samples using the QIAamp^®^ Fast DNA stool mini kit (Qiagen, Hilden, Germany) following the manufacturer's protocol. 16S rRNA genes were amplified using the universal primer set Bakt_805R (5′‐ GAC TAC HVG GGT ATC TAA TCC‐3′) and Bakt_341F (5′‐CCT ACG GGN GGC WGC AG‐3′) (Herlemann et al. 2011), obtained from MWG Eurofins, Germany.

For the polymerase chain reaction (PCR), a reaction mixture for each sample (final volume 50 *μ*L) was prepared in thin‐walled PCR tubes containing 31.75 *μ*L of DEPC‐treated water, 10 *μ*L of reaction buffer, 2 *μ*L of bovine serum albumin, 1 *μ*L of 25 mmol L^−1^ MgCl_2_, 1.25 *μ*L of each primer, 0.5 *μ*L of 100 mmol L^−1^ deoxynucleoside triphosphates, 0.25 *μ*L of Herculase II (Agilent, Waldbronn, Germany), and 2 *μ*L of template. The samples were preheated at 95°C for 4 min and then amplified in a thermal cycler (MyCycler; Bio‐Rad, Germany) under the following conditions: 28 cycles of denaturation at 95°C for 40 sec, annealing at 53°C for 40 sec, and elongation at 72°C for 1 min, followed by a final elongation step at 72°C for 7 min.

The PCR products were purified using the Agencourt AMPure XP–PCR purification kit (Beckman Coulter, Brea, CA, USA) following the manufacturer's instructions. The quality and yield of the DNA were subsequently determined in a PicoGreen^®^ dsDNA quantitation assay (protocol: “Quant‐iT^™^ PicoGreen^®^ dsDNA Reagent and Kits” from the manufacturer′s homepage) and by comparison with a calibration line obtained by measuring a serial dilution of DNA of known concentration (calf thymus DNA, Sigma‐Aldrich, Steinheim, Germany). A quantity of 2 *μ*L of each purified PCR sample was diluted in 8 *μ*L of Tris‐EDTA (TE) buffer and added to 90 *μ*L of PicoGreen^®^ working solution, consisting of the Quant‐iT^™^ PicoGreen^®^ dsDNA reagent (Eugene, OR, USA) and TE buffer (1:200). The absorbance of each sample was measured using a multimode microplate reader (Infinite^®^ M200 Pro; Tecan, Grödig/Salzburg, Austria). The 16S rDNA samples were then sent to Eurofins Genomics for 454 amplicon sequencing.

The SILVAngs data analysis service (Yilmaz et al. 2013) was used to align the resulting sequences with the SILVA incremental aligner (SINA) and to remove contaminations of the dataset by non‐rRNA sequences. SILVAngs performs an additional quality check based on a minimal length cutoff (50 bases) as well as ambiguity and homopolymer checks (max. 2%). After the quality control, identical reads were clustered according to 97% sequence identity operational taxonomic unit (OTUs) and on a per sample basis using cdhit‐version 3.1.2 (Li and Godzik 2006). For each OTU clustering, the longest read was then used as a reference of this cluster in a taxonomic classification using Basic Local Alignment Search Tool (BLAST); (National Center for Biotechnology Information, Bethesda MD, USA) (version 2.2.28+) in combination with the SILVA SSURef dataset (release 115). The resulting rank classification of the reference sequence of a cluster was mapped to all members of the respective cluster and to their replicates. Sequences having an average BLAST alignment coverage and alignment identity <93% were considered as unclassified. This method was first used by Klindworth et al. (2013) and Ionescu et al. (2012).

## Results

### Bacterial diversity based on 16S rRNA gene amplicon sequencing

**Figure 1:**
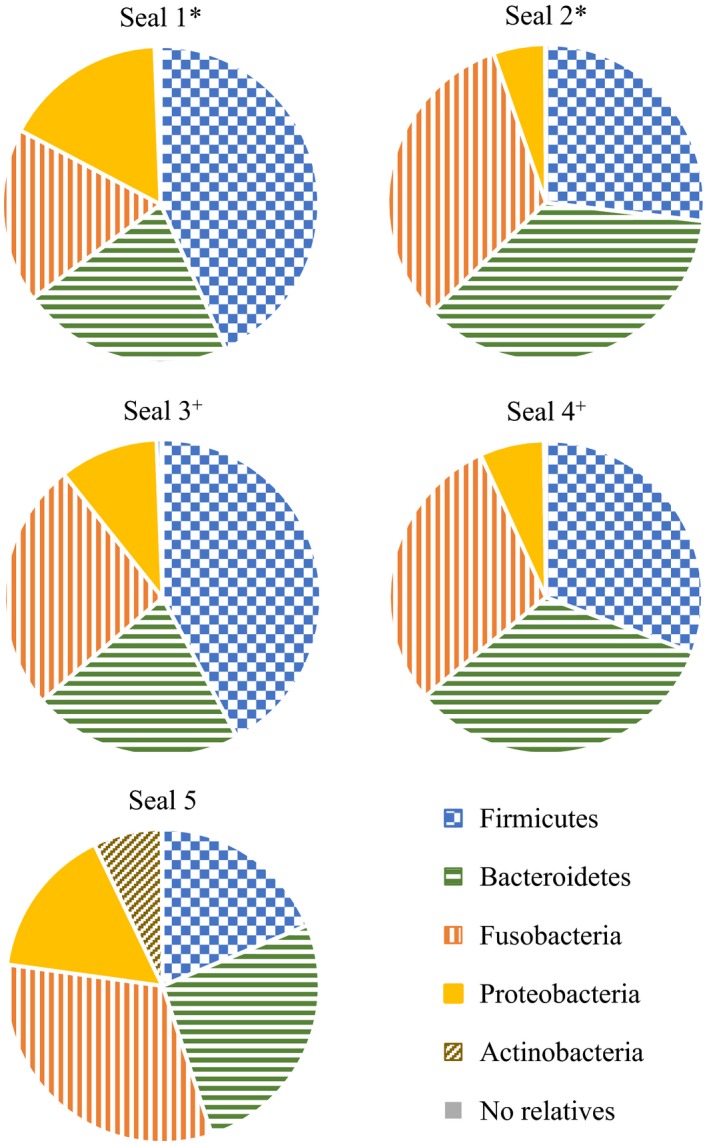
Percentage composition of the fecal bacterial community of the five investigated harbor seals (seals 1–5) on the phylum level, based on 454 pyrosequencing results. *,^+^ Half‐brothers (common father).

Amplicon sequencing of the 16S rRNA gene of all five seals yielded 22,058 reads (3466 for seal 1, 4374 for seal 2, 5531 for seal 3, 4207 for seal 4, and 4480 for seal 5), with 70 classified OTUs, mostly on the genus level and sum normalized for unbiased comparisons between samples. The dominant bacterial phyla based on amplicon sequencing of the feces of the harbor seals were *Firmicutes* (19–43%), *Bacteroidetes* (22–36%), *Fusobacteria* (18–32%), and *Proteobacteria* (5–17%) (Fig. 1). Other phyla found in some of the samples albeit to a lesser extent were *Actinobacteria, Chloroflexi*,* Fibrobacteres*,* Verrucomicrobia*, and Candidate Division OD1. Within the *Firmicutes*, the most diverse phylum, members of the genera *Oscillibacter* (0.4–12.8%), *Fecalibacterium* (0.6–12.7%), and *Clostridium* (0.04–5.9%) as well as representatives of the families *Ruminococcacae* (7.2–20.2%), *Peptostreptococcaceae* (0.2–4.6%), and *Erysipelotrichiaceae* (0.3–5.5%), were dominant and present in all five animals. *Bacteroidetes* were mainly represented by the genera *Bacteroides* (8.8–24.5%), *Alistipes* (3.6–10.4%), and *Prevotella* (0.9–7.3%). Within the *Proteobacteria*, the genera *Escherichia/Shigella* (0.2–9.4%), *Anaerobiospirillum* (0.1–5.4%), and *Sutterella* (1.3–2.5%) were detected in the five samples. The genus *Thalassospira* was only dominant in seal 5 (13.0%) and seal 3 (5.3%). *Actinobacteria*, represented by the genus *Collinsella* (7.1%), were only present in a larger proportion in seal 5. The dominant genus within the phylum *Fusobacteria* was *Fusobacterium* (17.8–32.3%), which was present in all five harbor seals (Table 2).

**Table 2:** Percentage of classified OTUs (OTU clustering 97%) in the fecal microbial communities of the five investigated harbor seals as determined by 454 pyrosequencing

Phylum	Genus/Family member	Seal 1*	Seal 2*	Seal 3^+^	Seal 4^+^	Seal 5
*Actinobacteria*	*Atopobium*	0	0.05	0.14	0	0
	*Collinsella*	0.09	0	0.33	0	7.05
*Bacteroidetes*	***Bacteroides***	**8.80**	**23.30**	**13.60**	**24.51**	**12.90**
	*Barnesiella*	0.20	0	0.04	0	0
	*Odoribacter*	0	0	0.78	0.16	0
	*Parabacteroides*	0.14	0	0.13	0.02	0
	***Prevotella***	6.32	7.30	0.90	4.89	2.79
	***Alistipes***	5.77	5.02	5.86	3.57	10.38
	**Member of the family ** ***S24‐7***	0.29	0.07	0.25	0.16	0.09
	Member of the family *WCHB1‐69*	0	0.21	0	0	0
*Firmicutes*	**Uncultured member of the family ** ***Christensenellaceae***	0.09	0.07	0.24	0.07	0.11
	***Clostridium***	2.51	2.19	0.04	5.92	1.18
	***Blautia***	0.32	0.10	0.25	0.23	0.09
	**Member ** ***inc. sed.*** **of the family ** ***Lachnospiraceae***	3.09	3.49	1.45	0.09	3.91
	**Uncultured member of the family ** ***Lachnospiraceae***	0.49	0.05	0.11	0.02	0.13
	**Member ** ***inc. sed.*** **of the family ** ***Peptostreptococcaceae***	1.38	4.56	0.24	4.09	0.98
	***Anaerotruncus***	0.58	0.26	0.18	0.07	0.31
	***Faecalibacterium***	3.03	1.31	12.73	0.59	2.03
	**Member ** ***inc. sed.*** **of the family ** ***Ruminococcaceae***	0.78	0.88	1.08	0.07	0.56
	***Oscillibacter***	12.81	3.78	1.92	1.12	0.36
	**Uncultured member of the family ** ***Ruminococcaceae***	11.60	7.18	20.23	17.99	7.57
	**Member ** ***inc. sed.*** **of the family ** ***Erysipelotrichaceae***	5.54	2.64	0.81	0.25	0.87
	Uncultured member of the family *Erysipelotrichaceae*	0.26	0.02	0	0	0
	***Phascolarctobacterium***	0.46	0.21	2.66	0.18	0.47
*Fusobacteria*	***Fusobacterium***	17.77	31.83	25.55	29.10	32.28
*Proteobacteria*	*Thalassospira*	0.14	0	5.28	0	12.99
	***Sutterella***	1.33	1.38	2.48	2.38	1.72
	*Sulfurimonas*	0	0	0	0	0.65
	***Anaerobiospirillum***	5.42	2.61	1.16	1.44	0.09
	Uncultured member of the family *Succinivibrionaceae*	0.12	0	0	0	0
	***Escherichia‐Shigella***	9.41	1.26	1.16	2.70	0.20
No relative		0.40	0.02	0.11	0.09	0.07
Percentage of core members		97.79	99.49	92.9	99.45	79.02

Only bacterial members with a relative abundance >0.1% are shown. *inc. sed*.: *incertae sedis* (of uncertain placement). Genus/family members shown in bold are among the 21 bacterial groups present in all five seals and considered to comprise the core members of their gut microbiota.

*,^+^Half‐brothers (common father).

### FISH analyses

**Figure 2:**
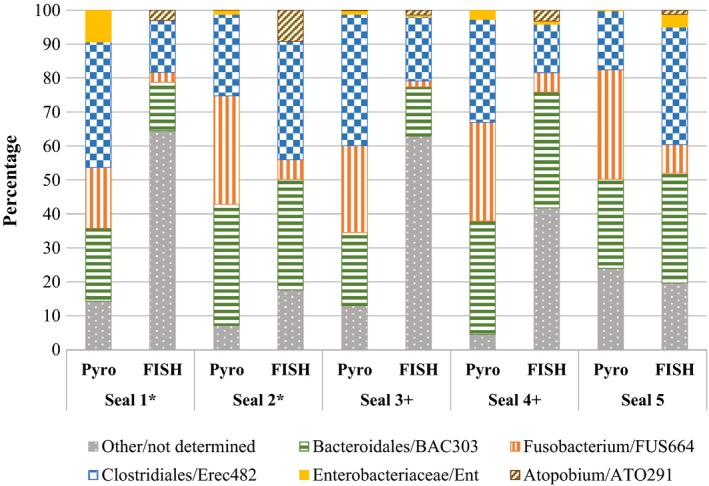
Comparison of the percentages of bacterial groups, as determined by fluorescence in situ hybridization (FISH) and 454 pyrosequencing data (pyro). *,^+^ Half‐brothers (common father).

The total hybridized bacterial cells from the five FISH probes accounted for 25–82% of the DAPI counts. The dominant bacterial groups were *Clostridiales* (Erec482), with 14–35% of the total DAPI counts, and *Bacteroidales* (BAC303), with 14–34%. Bacteria of the genus *Fusobacterium* (2–8%) were detected in all of the seals using the FUS664 probe. Members of the *Atopobium* cluster were also present in all five animals, with an abundance of 1% in the feces of seal 3 and seal 5, 3% in those of seal 1 and seal 4, and 9% in those of seal 2. The Ent probe, specific for *Enterobacteriaceae*, yielded positive results in seal 4 (1%), seal 3 (0.5%), and seal 5 (4%) (Fig. 2).

The percentages of cells detected by the FISH probes in relation to the DAPI counts and the percentages of the corresponding sequences obtained by amplicon sequencing are shown in Figure 2 for all seals. The genus *Fusobacterium* was more strongly represented in each seal by sequence data (17.8–32.3%) than by the FUS664 probe (1.7–8.4%). Compared to the sequence data, the percentages obtained with the Erec482 probe, which mainly hybridizes with bacteria of the order *Clostridiales*, were lower for seal 1 (15.2%), seal 3 (18.9%), and seal 4 (14.2%) and higher for seal 2 (34.9%) and seal 5 (34.6%). Positive results were obtained with the ATO291 probe, specific for the *Atopobium* cluster, in all individuals (1.3–9.2%), whereas according to 454 pyrosequencing *Atopobium* sequences were only present in seal 2 (0.05%) and seal 3 (0.2%). The Ent probe detected *Enterobacteriaceae* only in seal 3 (0.5%), seal 4 (1.0%), and seal 5 (3.6%), but based on the sequence data, *Enterobacteriaceae* were present in all individuals (0.2–9.4%). Comparable percentages were obtained for the order *Bacteroidales* detected by the BAC303 probe (14.2–34.1%) and by 454 pyrosequencing (21.6–35.7%).

### Comparison of the fecal bacterial communities of the five seals

Based on the OTU clustering, 21 bacterial groups were shared by all five harbor seals. These groups consisted of the genera *Bacteroides*,* Prevotella*,* Alistipes*,* Clostridium*,* Blautia*,* Anaerotruncus*,* Faecalibacterium*,* Oscillibacter*,* Phascolarctobacterium*,* Fusobacterium*,* Sutterella*,* Anaerobiospirillum*, and *Escherichia/Shigella*, and representatives of the families *S24‐7*,* Christensenellaceae*,* Lachnospiraceae*,* Peptostreptococcacaeae*,* Ruminococcaceae*, and *Erysipelotrichaceae* (Table 2). These shared bacterial groups can be seen as the core members of the five investigated harbor seals. They contributed an average of 93.7 + 8.7% to the total relative abundance of the fecal bacterial community (Table 2).

**Figure 3:**
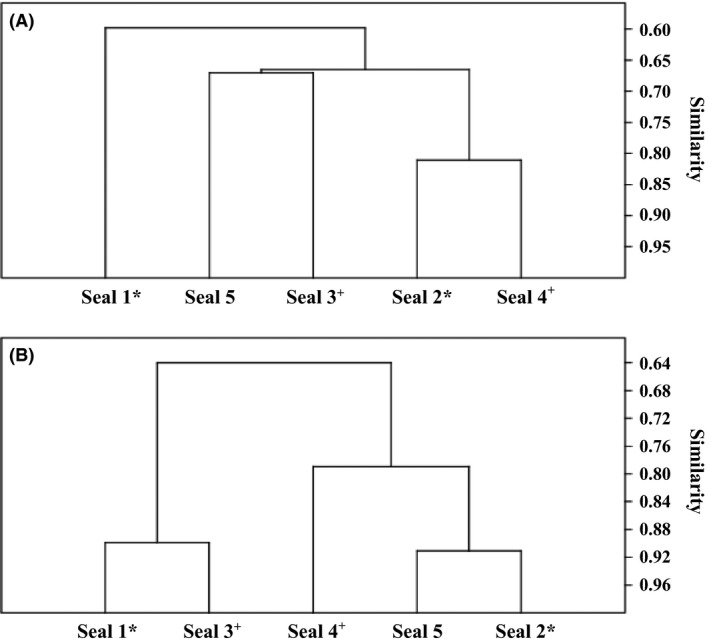
Hierarchical cluster analysis dendrograms of the fecal community of the five investigated harbor seals (seals 1–5) based on 454 pyrosequencing data (A) and fluorescence in situ hybridization (B). Cluster analysis was performed using the Past 3.02a software. Algorithm, unweighted pair‐group average (UPGMA); similarity index, Bray‐Curtis index. *,^+^ Half‐brothers (common father).

Two cluster analyses were performed based on the data obtained by FISH analyses and amplicon sequencing (Fig. 3). Different clusters were identified in the two dendrograms; however, both cluster analyses showed greater similarity in the bacterial communities of the feces of unrelated than of related seals.

## Discussion

Among the novel aspects of this study was the microbiological analyses of fresh feces collected from pinnipeds administered with enema. This was possible because the seals are tame and well trained. In previous studies of the gut microbiota of pinnipeds, fecal material was obtained by rectal swabbing of sedated animals (Nelson et al. 2013a) or scooping the material from the cloaca (Smith et al. 2013). In another study, the colon contents of culled seals were analyzed (Glad et al. 2010).

The samples were assessed using two different methods, FISH analyses and sequencing, but their results were largely comparable. However, the two methods differ in their advantages and disadvantages and are based on different approaches. FISH analyses allow the quantification of bacterial groups based on cell enumeration, but diversity determinations are limited by the selection of specific probes. In addition, there may be false‐positive and false‐negative results depending on the probes. Sequence data reveal most of the diversity, but the abundances are only relative and the number of reads are vulnerable to PCR bias and/or differences in the copy numbers of 16S rRNA genes (Farrelly et al. 1995). In this study, the percentages of bacterial groups were mostly higher in the sequence analysis than in the FISH study **(**Fig. 2 **),** which may have been due to a lack of coverage by the FISH probes (Table 3). For example, probe FUS664 covers only 84.2% of the genus *Fusobacterium*. Conversely, the higher percentages revealed by FISH analyses may have been the result of outgroup matches (Table 3) or differences in the enzymatic treatment of samples, as in the case of the genus *Atopobium*. In the FISH analysis using the ATO291 probe, pretreatment with lysozyme and achromopeptidase was needed to improve cell permeabilization, as also shown by Sekar et al. (2003). Thus, amplicon sequencing may have underestimated the percentage abundance of the genus *Atopobium* because of insufficient cell permeabilization or lysis.

**Table 3:** Coverage and specificity of the probes used in this study and previously checked using TestProbe 3.0 of the SILVA database [http://www.arb-silva.de/search/testprobe/, accessed on 08/11/2015, (Quast et al. 2013)]

Probe	Bacterial group	Coverage (%)	Specificity (%)	Outgroup matches
ATO291	*Atopobium*	92	99.9	600
BAC303	*Bacteroidales*	50.6	100	186
Ent	*Enterobacteriaceae*	71.3	99.9	571
Erec482	*Clostridiales*	36.9	100	225
FUS664	*Fusobacterium*	84.2	100	48

The dominant bacterial phyla in the feces of the investigated harbor seals, as revealed by 454 amplicon sequencing, were *Firmicutes* (19–43%), *Bacteroidetes* (22–36%), *Fusobacteria* (18–32%), and *Proteobacteria* (5–17%) (Fig. 1). In some of the seals, the phyla *Actinobacteria, Chloroflexi*,* Fibrobacteres*,* Verrucomicrobia*, and Candidate Division OD1 were additionally present, but the percentages were low. In a previous study, in which the colon contents of a wild male harbor seal from the coast of Northern Norway were investigated with respect to the bacterial community (Kristiansen 2007; Glad et al. 2010), *Firmicutes* (all belonging to *Clostridiales*), *Bacteroidetes* (all belonging to *Bacteroidales*), and *Fusobacteria* (all belonging to *Fusobacteriales*) were detected in relative abundances of 49.4%, 49.4%, and 1.2%, respectively (Kristiansen 2007). The diversity in the fecal flora of the harbor seals from this study was higher, perhaps because of the greater number of opportunities for bacterial transmission afforded by the seals' constant social interactions, co‐habitation, and interactions with human keepers as well as with the general public, as recently shown for wild and captive leopard seals (Nelson et al. 2013a).

A comparison of the gut microbiota of conspecifics or related species can provide information on influencing factors such as diet but also identify a unique core community of bacteria, which may be the result of co‐evolution with the host. The core community in the five investigated harbor seals consisted of 21 shared bacterial groups (Table 2). Based on previous studies of other phocid seals, including hooded seals, gray seals, southern elephant seals, and leopard seals, and as proposed by Nelson (2012), the following genera can be considered as comprising the core gut microbiota of phocid seals: *Bacteroides*,* Faecalibacterium*,* Fusobacterium*,* Oscillibacter*,* Alistipes*,* Sutterella*,* Escherichia*‐*Shigella*,* Anaerobiospirillum*,* Clostridium*, and *Blautia* (Glad et al. 2010; Nelson 2012; Nelson et al. 2013a). Nelson (2012) suggested that these shared bacterial members may be seal‐specific and have co‐evolved with their hosts in accordance with their geographic separation, which implies differences in their diets. The core community might be linked to general functions, such as host immunity, so that its vertical transmission (mother‐to‐infant) ensures the maintenance of these bacteria. The functionality of the core members as an evolutionary trait will best be elucidated by studying related species from both captive and wild environments.

The relatively high degree of similarity in the fecal community of the investigated harbor seals may at least in part be due to the fact that they are fed the same diet and have the same environmental exposure, and/or to their constant and similar social interactions; host phylogeny may also play a role (e.g., Ley et al. 2008a,b; Yildirim et al. 2010; Nelson 2012). On the other hand, factors leading to differences in the gut microbiota of these harbor seals may have been due to differences in the preferred prey species, the different mothers and therefore different initial sources of intestinal bacteria, and varying antibiotic exposure. None of these could be tested in this study, as it would have required more controlled conditions. Furthermore, the differences may also have been by chance, for example, during the (initial) colonization of the gastrointestinal tract.

Among the five seals, there were two half‐brother pairs, each sharing a common father. This allowed consideration of genotype or relatedness as the influencing factor in shaping the gut microbiota without the interference of maternal influences, which could have masked the effect of genotype. Although previous studies have reported that host genotype influences the composition of the gut microbiota (e.g., Zoetendal et al. 2001; Stewart et al. 2005; Kovaks et al. 2011), the fecal communities of related seals were clearly not more similar than those of unrelated ones (Fig. 3). Due to the small sample size, further studies including half‐brothers or ‐sisters sharing a common father are necessary. However, it might also be possible that external factors, such as diet, antibiotic administration, and environmental exposure, may be stronger drivers than genotype or, at least in the investigated harbor seals, may have masked the effect of relatedness. The higher similarity of related individuals determined in previous studies (e.g., Zoetendal et al. 2001; Benson et al. 2010; Kovaks et al. 2011) can also be explained by maternal influences other than genotype, such as similar environmental and dietary influences or inoculation of the intestinal tract during passage of the infant through the birth canal (e.g., Bettelheim et al. 1974; Long and Swenson 1977; Mändar and Mikelsaar 1996; Ley et al. 2005). Mändar and Mikelsaar (1996) found similar prevailing microbes in the mother's vagina and her neonate's initial microbial community and that the predominance pattern of the mother's genital microorganisms significantly influenced the initial microecological relations of her newborn. Further investigations on the role of genotype in determining the gut microbiota should include studies on differently related conspecifics living separately and unrelated conspecifics living in the same environment and with a similar lifestyle (e.g., diet).

## Conclusion

This study evaluated the bacterial diversity in the feces of five harbor seals. The dominant bacterial phyla were *Firmicutes* (19–43%), *Bacteroidetes* (22–36%), *Fusobacteria* (18–32%), and *Proteobacteria* (5–17%). A core community, with a relatively high similarity among the five seals, consisted of 21 shared bacterial members that contributed an average relative abundance of 93.7 + 8.7% of the total fecal community. However, in contrast to previous studies, we found a higher similarity between unrelated than related seals. Future studies focusing on the occurrence, diversity, and functionality of the core members shared within a species and with related species will provide insights into the co‐evolution of bacteria and their hosts.

## Ethics statement

The experiments were carried out in accordance with the European Communities Council Directive of 24 November 1986 (86/609/EEC). According to §8 of the German Animal Welfare Act of 18 May 2006 (BGB I. I S. 1206, 1313), experiments conducted in this study were not subject to approval or notification, since they did not cause pain, suffering, or injuries to the animals.

## Conflict of Interest

The authors confirm that they have no conflicts of interest related to the content of this article.

## References

[mbo3369-bib-0001] Ambrose, N. S. , M. Johnson , D. W. Burdon , and M. R. B. Keighley . 1985 The influence of single dose intravenous antibiotics on faecal flora and emergence of *Clostridium difficile* . J. Antimicrob. Chemother. 15:319–326. 3846592 10.1093/jac/15.3.319

[mbo3369-bib-0002] Bailey, M. T. , S. E. Dowd , J. D. Galley , A. R. Hufnagle , R. G. Allen , and M. Lyte . 2011 Exposure to a social stressor alters the structure of the intestinal microbiota: implications for stressor‐induced immunomodulation. Brain Behav. Immun. 25:397–407. 21040780 10.1016/j.bbi.2010.10.023 PMC3039072

[mbo3369-bib-0003] Benson, A. K. , S. A. Kelly , R. Legge , F. Ma , S. J. Low , J. Kim , et al. 2010 Individuality in gut microbiota composition is a complex polygenic trait shaped by multiple environmental and host genetic factors. PNAS 107:18933–18938. 20937875 10.1073/pnas.1007028107 PMC2973891

[mbo3369-bib-0004] Bergman, E. N. , R. S. Reid , M. G. Murray , J. M. Brockway , and F. G. Whitelaw . 1965 Interconversions and production of volatile fatty acids in the sheep rumen. Biochem. J. 97:53–58. 16749123 10.1042/bj0970053 PMC1264542

[mbo3369-bib-0005] Bettelheim, K. A. , A. Breadon , M. C. Faiers , S. O'Farrell , and R. A. Shooter . 1974 The origin of O serotypes of *Escherichia coli* in babies after normal delivery. J. Hyg. (Lond) 72:67–70. 4593741 10.1017/s0022172400023226 PMC2130250

[mbo3369-bib-0006] Castillo, M. , S. M. Martín‐Orúe , M. Anguita , J. F. Pérez , and J. Gasa . 2007 Adaptation of gut microbiota to corn physical structure and different types of dietary fibre. Livestock Sci. 109(1–3):149–152.

[mbo3369-bib-0007] Cebra, J. J. 1999 Influences of microbiota on intestinal immune system development. Am. J. Clin. Nut. 69:1046–1051. 10.1093/ajcn/69.5.1046s 10232647

[mbo3369-bib-0008] Claesson, M. J. , I. B. Jeffery , S. Conde , S. E. Power , E. M. O'Connor , S. Cusack , et al. 2012 Gut microbiota composition correlates with diet and health in the elderly. Nature 488:178–184. 22797518 10.1038/nature11319

[mbo3369-bib-0009] De Filippo, C. , D. Cavalieri , M. Di Paola , M. Ramazzotti , J. B. Poullet , S. Massart , et al. 2010 Impact of diet in shaping gut microbiota revealed by a comparative study in children from Europe and rural Africa. PNAS 107:14691–14696. 20679230 10.1073/pnas.1005963107 PMC2930426

[mbo3369-bib-0010] Dethlefsen, L. , and D. A. Relman . 2011 Incomplete recovery and individualized responses of the human distal gut microbiota to repeated antibiotic perturbation. PNAS 108:4554–4561. 20847294 10.1073/pnas.1000087107 PMC3063582

[mbo3369-bib-0011] Dethlefsen, L. , S. Huse , M. L. Sogin , and D. A. Relman . 2008 The pervasive effects of an antibiotic on the human gut microbiota, as revealed by deep 16S rRNA sequencing. PLoS Biol. 6:2383–2400. 10.1371/journal.pbio.0060280 PMC2586385 19018661

[mbo3369-bib-0012] Farrelly, V. , F. A. Rainey , and E. Stackebrandt . 1995 Effect of genome size and rrn gene copy number on PCR amplification of 16S rRNA genes from a mixture of bacterial species. Appl. Environ. Microbiol. 61:2798–2801. 7618894 10.1128/aem.61.7.2798-2801.1995 PMC167554

[mbo3369-bib-0013] Franks, A. H. , H. J. M. Harmsen , G. C. Raangs , G. J. Jansen , F. Schut , and G. W. Welling . 1998 Variations of bacterial populations in human feces measured by fluorescent in situ hybridization with group‐specific 16S rRNA‐targeted oligonucleotide probes. Appl. Environ. Microbiol. 64:3336–3345. 9726880 10.1128/aem.64.9.3336-3345.1998 PMC106730

[mbo3369-bib-0014] Gill, S. R. , M. Pop , R. T. DeBoy , P. B. Eckburg , P. J. Turnbaugh , B. S. Samuel , et al. 2006 Metagenomic analysis of the human distal gut microbiome. Science 312:1355–1359. 16741115 10.1126/science.1124234 PMC3027896

[mbo3369-bib-0015] Glad, T. , V. F. Kristiansen , K. M. Nielsen , L. Brusetti , A. G. Wright , and M. A. Sundset . 2010 Ecological characterisation of the colonic microbiota in Arctic and Sub‐Arctic seals. Microb. Ecol. 60:320–330. 20523986 10.1007/s00248-010-9690-x

[mbo3369-bib-0016] Glöckner, F. O. , R. Amann , A. Alfreider , J. Pernthaler , R. Psenner , K. Trebesius , et al. 1996 An in situ hybridization protocol for detection and identification of planktonic bacteria. Syst. Appl. Microbiol. 19:403–406.

[mbo3369-bib-0017] Gorbach, S. L. , M. Barza , M. Giuliano , and N. V. Jacobus . 1988 Colonization resistance of the human intestinal microflora: testing the hypothesis in normal volunteers. Eur. J. Clin. Microbiol. Infect. Dis. 7:98–102. 3132394 10.1007/BF01962192

[mbo3369-bib-0018] Harmsen, H. J. M. , A. C. M. Wildeboer‐Veloo , J. Grijpstra , J. Knol , J. E. Degener , and G. W. Welling . 2000 Development of 16S rRNA‐based probes for the *Coriobacterium* group and the *Atopobium* cluster and their application for enumeration of *Coriobacteriaceae* in human feces from volunteers of different age groups. Appl. Environ. Microbiol. 66:4523–4527. 11010909 10.1128/aem.66.10.4523-4527.2000 PMC92335

[mbo3369-bib-0019] He, X. , Y. Tian , L. Guo , R. Lux , D. R. Zusman , and W. Shi . 2010 Oral‐derived bacterial flora defends its domain by recognizing and killing intruders ‐ A molecular analysis using *Escherichia coli* as a model intestinal bacterium. Microb. Ecol. 60:655–664. 20625713 10.1007/s00248-010-9708-4 PMC2954290

[mbo3369-bib-0020] Herlemann, D. P. R. , M. Labrenz , K. Jürgens , S. Bertilsson , J. J. Waniek , and A. F. Andersson . 2011 Transitions in bacterial community along the 2000 km salinity gradient of the Baltic Sea. ISME J. 5:1571–1579. 21472016 10.1038/ismej.2011.41 PMC3176514

[mbo3369-bib-0021] Hume, F. , M. A. Hindell , D. Pemberton , and R. Gales . 2004 Spatial and temporal variation in the diet of a high trophic level predator, the Australian fur seal (*Arctocephalus pusillus doriferus*).Maine. Biology 144:407–415.

[mbo3369-bib-0022] Ionescu, D. , C. Siebert , L. Polerecky , Y. Y. Munwes , C. Lott , S. Häusler , et al. 2012 Microbial and chemical characterization of underwater fresh water springs in the Dead Sea. PLoS ONE 7:e38319. 22679498 10.1371/journal.pone.0038319 PMC3367964

[mbo3369-bib-0023] Ivanov, I. I. , R. De Llanos Frutos , N. Manel , K. Yoshinaga , D. B. Rifkin , R. B. Sartor , et al. 2008 Specific microbiota direct the differentiation of IL‐17‐producing T‐helper cells in the mucosa of the small intestine. Cell Host Microbe 4:337–349. 18854238 10.1016/j.chom.2008.09.009 PMC2597589

[mbo3369-bib-0024] Kempf, V. A. J. , K. Trebesius , and I. B. Autenrieth . 2000 Fluorescent in situ hybridization allows rapid identification of microorganisms in blood cultures. J. Clin. Microbiol. 38:830–838. 10655393 10.1128/jcm.38.2.830-838.2000 PMC86216

[mbo3369-bib-0025] Klindworth, A. , E. Pruesse , T. Schweer , J. Peplies , C. Quast , M. Horn , et al. 2013 Evaluation of general 16S ribosomal RNA gene PCR primers for classical and next‐generation sequencing‐based diversity studies. Nucleic Acids Res. 41:e1. 22933715 10.1093/nar/gks808 PMC3592464

[mbo3369-bib-0026] Kovaks, A. , N. Ben‐Jacob , H. Tayem , E. Halperin , F. A. Iraqi , and U. Gophna . 2011 Genotype is a stronger determinant than sex of the mouse gut microbiota. Microb. Ecol. 61:423–428. 21181142 10.1007/s00248-010-9787-2

[mbo3369-bib-0027] Kristiansen, V. F . 2007 Ampicillin resistance and bacterial diversity in colon content from grey seals (Halichoerus grypus) and harbour seals (Phoca vitulina) at the coast of northern Norway. (Master Thesis), University of TromsØ, Norway.

[mbo3369-bib-0028] Lavery, T. J. , B. Roudnew , J. Seymour , J. G. Mitchell , and T. Jeffries . 2012 High nutrient transport and cycling potential revealed in the microbial metagenome of Australian sea lion (*Neophoca cinerea*) faeces. PLoS ONE 7:e36478. 22606263 10.1371/journal.pone.0036478 PMC3350522

[mbo3369-bib-0029] Leser, T. D. , and L. Molbak . 2009 Better living through microbial action: the benefits of the mammalian gastrointestinal microbiota on the host. Environ. Microbiol. 11:2194–2206. 19737302 10.1111/j.1462-2920.2009.01941.x

[mbo3369-bib-0030] Ley, R. E. , F. Bäckhed , P. Turnbaugh , C. A. Lozupone , R. D. Knight , and J. I. Gordon . 2005 Obesity alters gut microbial ecology. PNAS 102:11070–11075. 16033867 10.1073/pnas.0504978102 PMC1176910

[mbo3369-bib-0031] Ley, R. E. , M. Hamady , C. Lozupone , P. Turnbaugh , R. R. Ramey , S. Bircher , et al. 2008a Evolution of mammals and their gut microbes. Science 320:1647–1651. 18497261 10.1126/science.1155725 PMC2649005

[mbo3369-bib-0032] Ley, R. E. , C. Lozupone , M. Hamady , R. Knight , and J. I. Gordon . 2008b Worlds within worlds: evolution of the vertebrate gut microbiota. Nat. Rev. Microbiol. 6:776–788. 18794915 10.1038/nrmicro1978 PMC2664199

[mbo3369-bib-0033] Li, W. , and A. Godzik . 2006 Cd‐hit: a fast program for clustering and comparing large sets of protein or nucleotide sequences. Bioinformatics 22:1658–1659. 16731699 10.1093/bioinformatics/btl158

[mbo3369-bib-0034] Lombardo, M. P. 2008 Access to mutualistic endosymbiotic microbes: an underappreciated benefit of group living. Behav. Ecol. Sociobiol. 62:479–497.

[mbo3369-bib-0035] Long, S. S. , and R. M. Swenson . 1977 Development of anaerobic fecal flora in healthy newborn infants. J. Pediatr. 91:298–301. 874690 10.1016/s0022-3476(77)80836-6

[mbo3369-bib-0036] Loy, A. , F. Maixner , M. Wagner , and M. Horn . 2007 probeBase ‐ an online resource for rRNA‐targeted oligonucleotide probes: new features 2007. Nucleic Acids Res. 35(Database issue): D800–D804. 17099228 10.1093/nar/gkl856 PMC1669758

[mbo3369-bib-0037] Mändar, R. , and M. Mikelsaar . 1996 Transmission of mother′s microflora to the newborn at birth. Biol. Neonate 69:30–35. 8777246 10.1159/000244275

[mbo3369-bib-0038] Manz, W. , R. Amann , W. Ludwig , M. Vancanneyt , and K. Schleifer . 1996 Application of a suite of 16S rRNA‐specific oligonucleotide probes designed to investigate bacteria of the phylum *cytophaga‐flavobacter‐bacteroides* in the natural environment. Microbiology 142:1097–1106. 8704951 10.1099/13500872-142-5-1097

[mbo3369-bib-0039] Mazmanian, S. K. , C. H. Liu , A. O. Tzianabos , and D. L. Kasper . 2005 An immunomodulatory molecule of symbiotic bacteria directs maturation of the host immune system. Cell 122:107–118. 16009137 10.1016/j.cell.2005.05.007

[mbo3369-bib-0040] Mueller, S. , K. Saunier , C. Hanisch , E. Norin , L. Alm , T. Midtvedt , et al. 2006 Differences in fecal microbiota in different European study populations in relation to age, gender, and country: a cross‐sectional study. Appl. Environ. Microbiol. 72:1027–1033. 16461645 10.1128/AEM.72.2.1027-1033.2006 PMC1392899

[mbo3369-bib-0041] Nalepa, C. A. , D. E. Bignell , and C. Bandi . 2001 Detritivory, coprophagy, and the evolution of digestive mutualisms in *Dictyoptera* . Insectes Soc. 48:194–201.

[mbo3369-bib-0042] Nelson, T . 2012 Factors influencing the gut microbiota of Antarctic seals. [Ph. D. Thesis], University of New South Wales, Sydney, Australia

[mbo3369-bib-0043] Nelson, T. M. , T. L. Rogers , A. R. Carlini , and M. V. Brown . 2013 Diet and phylogeny shape the gut microbiota pf antarctic seals: a comparison of wild and captive animals. Environ. Microbiol. 15:1132–1146. 23145888 10.1111/1462-2920.12022

[mbo3369-bib-0044] Parker, D. S. 1976 The measurement of production rates of volatile fatty acids in the caecum of the conscious rabbit. Br. J. Nutr. 36:61–70. 949469 10.1079/bjn19760058

[mbo3369-bib-0045] Quast, C. , E. Pruesse , P. Yilmaz , J. Gerken , T. Schweer , P. Yarza , et al. 2013 The SILVA ribosomal RNA gene database project: improved data processing and web‐based tools. Nucleic Acids Res. 41(Database issue): D590–D596. 23193283 10.1093/nar/gks1219 PMC3531112

[mbo3369-bib-0046] Ramotar, K. , J. M. Conly , and T. J. Louie . 1984 Production of menaquinones by intestinal anaerobes. J. Infect. Dis. 150:213–218. 6470528 10.1093/infdis/150.2.213

[mbo3369-bib-0047] Sekar, R. , A. Pernthaler , J. Pernthaler , F. Warnecke , T. Posch , and R. Amann . 2003 An improved protocol for quantification of freshwater *Actinobacteria* by fluorescence in situ hybridization. Appl. Environ. Microbiol. 69:2928–2935. 12732568 10.1128/AEM.69.5.2928-2935.2003 PMC154517

[mbo3369-bib-0048] Smith, S. C. , A. Chalker , M. L. Dewar , and J. P. Y. Amould . 2013 Age‐related differences revealed in Australian fur seal *Arctocephalus pusillus doriferus* gut microbiota. FEMS Microbiol. Ecol. 86:1–10. 23746080 10.1111/1574-6941.12157

[mbo3369-bib-0049] Staniland, I. J. , and R. I. Taylor . 2003 An enema method for obtaining fecal material from known individual seals on land. Mar. Mammal Sci. 19:363–370.

[mbo3369-bib-0050] Stewart, J. A. , V. S. Chadwick , and A. Murray . 2005 Investigations into the influence of host genetics on the predominant eubacteria in the faecal microflora of children. J. Med. Microbiol. 54(Pt 12):1239–1242. 16278440 10.1099/jmm.0.46189-0

[mbo3369-bib-0051] Thompson, C. L. , B. Wang , and A. J. Holmes . 2008 The immediate environment during postnatal development has long‐term impact on gut community structure in pigs. ISME J. 2:739–748. 18356821 10.1038/ismej.2008.29

[mbo3369-bib-0052] Thurnheer, T. , R. Gmür , and B. Guggenheim . 2004 Multiplex FISH analysis of a six‐species bacterial biofilm. J. Microbiol. Methods 56:37–47. 14706749 10.1016/j.mimet.2003.09.003

[mbo3369-bib-0053] Troyer, K. 1984 Microbes, herbivory and the evolution of social behavior. J. Theor. Biol. 106:157–169.

[mbo3369-bib-0054] Turnbaugh, P. J. , R. E. Ley , M. A. Mahowald , V. Magrini , E. R. Mardis , and J. I. Gordon . 2006 An obesity‐associated gut microbiome with increased capacity for energy harvest. Nature 444:1027–1031. 17183312 10.1038/nature05414

[mbo3369-bib-0055] Turnbaugh, P. J. , M. Hamady , T. Yatsunenko , B. L. Cantarel , A. Duncan , R. E. Ley , et al. 2009 A core gut microbiome in obese and lean twins. Nature 457:480–485. 19043404 10.1038/nature07540 PMC2677729

[mbo3369-bib-0056] Van den Bogaard, A. E. J. M. , W. F. Weidema , C. P. A. Van Boven , and D. Van der Waay . 1986 Recolonization and colonization resistance of the large bowel after three methods of preoperative preparation of the gastrointestinal tract for elective colorectal surgery. J. Hyg. (Lond.) 97:49–59. 3734441 10.1017/s0022172400064342 PMC2082873

[mbo3369-bib-0057] Van der Waay, D. , J. M. Berghuis‐De Vries , and J. E. C. Lekkerkerk‐Van der Wees . 1971 Colonization resistance of the digestive tract in conventional and antibiotic‐treated mice. J. Hyg. (Lond.) 69:405–411. 4999450 10.1017/s0022172400021653 PMC2130899

[mbo3369-bib-0058] Yildirim, S. , C. J. Yeoman , M. Sipos , M. Torralba , B. A. Wilson , T. L. Goldberg , et al. 2010 Characterization of the fecal microbiome from non‐human wild primates reveals species specific microbial communities. PLoS ONE 5:1–11. 10.1371/journal.pone.0013963 PMC2980488 21103066

[mbo3369-bib-0059] Yilmaz, P. , L. W. Parfrey , P. Yarza , J. Gerken , E. Pruesse , C. Quast , et al. 2013 The SILVA and All‐species Living Tree Project (LTP) taxonomic frameworks. Nucleic Acids Res. 42:D643–D648. 24293649 10.1093/nar/gkt1209 PMC3965112

[mbo3369-bib-0060] Zhang, H. , J. K. DiBaise , A. Zuccolo , D. Kudrna , M. Braidotti , Y. Yu , et al. 2008 Human gut microbiota in obesity and after gastric bypass. PNAS 106:2365–2370. 10.1073/pnas.0812600106 PMC2629490 19164560

[mbo3369-bib-0061] Zoetendal, E. G. , A. D. L. Akkermans , W. M. Akkermans‐van Vliet , J. A. G. M. De Visser , and W. M. De Vos . 2001 The host genotype affects the bacterial community in the human gastrointestinal tract. Microb. Ecol. Health Dis. 13:129–134.

